# Specific autoantigens identified by sera obtained from mice that are immunized with testicular germ cells alone

**DOI:** 10.1038/srep35599

**Published:** 2016-10-18

**Authors:** Hayato Terayama, Shuichi Hirai, Munekazu Naito, Ning Qu, Chiaki Katagiri, Kenta Nagahori, Shogo Hayashi, Hiraku Sasaki, Shota Moriya, Masaki Hiramoto, Keisuke Miyazawa, Naoyuki Hatayama, Zhong-Lian Li, Kou Sakabe, Masayuki Matsushita, Masahiro Itoh

**Affiliations:** 1 Department of Anatomy, Division of Basic Medical Science, Tokai University School of Medicine, Kanagawa, Japan; 2 Department of Anatomy, Tokyo Medical University, Tokyo, Japan; 3 Department of Anatomy, Aichi Medical University, Aichi, Japan; 4 Department of Molecular and Cellular Physiology, Graduate School of Medicine, University of the Ryukyus, Okinawa, Japan; 5 Department of Health Science, School of Health and Sports Science, Juntendo University, Chiba, Japan; 6 Department of Biochemistry, Tokyo Medical University, Tokyo, Japan

## Abstract

There are various autoimmunogenic antigens (AIs) in testicular germ cells (TGCs) recognized as foreign by the body’s immune system. However, there is little information of TGC-specific AIs being available. The aim of this study is to identify TGC-specific AIs. We have previously established that immunization using viable syngeneic TGC can also induce murine experimental autoimmune orchitis (EAO) without using any adjuvant. This study is to identify TGC-specific AIs by TGC liquid chromatography–tandem mass spectrometry analysis, followed by two-dimensional gel electrophoresis that reacted with serum IgG from EAO mice. In this study, we identified 11 TGC-specific AIs that reacted with serum from EAO mice. Real-time RT-PCR analysis showed that the mRNA expressions of seven TGC-specific AIs were significantly higher in only mature testis compared to other organs. Moreover, the recombinant proteins of identified 10 (except unnamed protein) TGC-specific AIs were created by using human embryonic kidney 293 (HEK293) cells and these antigencities were reconfirmed by Western blot using EAO serum reaction. These results indicated Atp6v1a, Hsc70t, Fbp1 and Dazap1 were candidates for TGC-specific AIs. Identification of these AIs will facilitate new approaches for understanding infertility and cancer pathogenesis and may provide a basis for the development of novel therapies.

Spermatogenesis is the primary biological process occurring in the testis and produces mature haploid spermatozoa from diploid spermatogonia. This developmental process is complicated and involves a series of cell differentiation and biological events including spermatogonial proliferation, spermatocyte meiosis, and morphological changes of rounde spermatid^1^,^2^. Elucidation of the molecular mechanisms underlying spermatogenesis is important for understanding the genetic regulation of normal male germ cell development. This understanding can also direct strategies for the clinical diagnosis and treatment of male infertility. Therefore, investigation of the molecular mechanisms of testis development and spermatogenesis are prominent areas of research in the field of reproductive biology.

The testis is known as an immunologically privileged organ. Immune tolerance has already been established at birth when testicular germ cells (TGC) contain only stem cells or spermatogonia^3^,^4^. After puberty, they differentiate into spermatocytes and spermatids; the differentiation involves the expression of new molecules as spermatogenesis begins. Therefore, TGC are believed to contain various cell type-specific autoantigens which are recognized as foreign by the immune system^3^,^4^. The blood–testis barrier (BTB), formed by Sertoli cells, protects autoimmunogeneic TGC from any autoimmune attack^3^,^4^. Moreover, testicular cells express and secrete numerous immunoregulatory molecules that have important roles in the regulation of immune responses in the testes. These molecules create a regulatory system called “testicular immune privilege” and include androgens, activin, Fas ligand, protein S, and immunosuppressive cytokines such as interleukin (IL)-10, IL-35 and transforming growth factor (TGF)-β^5^,^6^,^7^,^8^,^9^,^10^,^11^,^12^. When the BTB is functionally damaged, TGC autoantigens can pass beyond the seminiferous epithelium and create a continuous stream of AIs that are exposed to systemic immune system, often for extended periods of time^3^. For example, damage to BTB of testis due to infection, biopsy, torsion, or surgery in the scrotal area induces orchitis in the contralateral testis^13^,^14^,^15^. Therefore, the AIs in TGC can be considered a critical target of autoimmune damage.

Recent studies have demonstrated that testicular inflammatory disorders leading to impairment of spermatogenesis are an important cause for male infertility, and autoimmune orchitis is noticed as one of main reasons^16^,^17^,^18^. Experimental autoimmune orchitis (EAO) is a model of chronic testicular inflammation resulting in male infertility^3^,^4^,^19^,^20^,^21^,^22^. The pathological condition is characterized by T-cell-dependent lymphocytic inflammation and damage to the seminiferous tubules involving the shedding and apoptosis of germ cells^3^,^4^,^19^,^20^,^21^,^22^,^23^,^24^,^25^,^26^,^27^,^28^. In rats and mice, EAO is classically induced by immunization with testicular homogenate (TH) plus complete Freund’s adjuvant (CFA) and *Bordetella pertussis* (BP); it is thought that treatment with the two adjuvants is required to enhance immune responses, resulting in the breakdown of testicular immune privilege^27^,^29^,^30^. We have recently reported that CFA and BP treatment alone augments autoimmune reactions against some testicular autoantigens^31^. These results indicate that the treatment with adjuvants alone can evoke autoimmune reactions against some AIs irrespectively with exposure to TH^31^. We have previously established another EAO model induced in both A/J and C3H/He mice with a very high incidence by two subcutaneous injections of viable syngeneic TGC without using any adjuvant^21^. Our EAO model is unique because serum autoantibodies are only against acrosomal regions of sperm and spermatids, but not Sertoli cells, Leydig cells and seminiferous tubular basement membrane^3^,^25^,^26^,^32^. This model showed that the immunologically privileged state of the testis is easily overcome using only two TGC injections.

On the other hand, TGC-specific AIs have also received considerable attention because of their role as cancer/testis antigens (CTs)^33^,^34^. CTs are protein antigens with expression normally restricted to adult TGCs and yet they become aberrantly activated in and expressed by a proportion of various types of cancer, including melanoma, lung cancer, and pancreatic cancer^33^,^34^,^35^. Hence, CTs are promising candidates for cancer immunotherapy targets and have become a major focus of vaccine-based clinical trials in recent years. Thus, information on the testis-specific proteins and proteins expressed after puberty may reveal additional biomarker candidates for cancer diagnosis/prognosis.

Previous studies using TH + CFA + BP-induced EAO rat and vasectomized mouse models have demonstrated that the proteins endoplasmic reticulum 60, heat shock protein 70, a partial region of D3p domain of Zan with B cell epitope, and others are AIs that are involved in testicular autoimmune response^36^,^37^. However, there is currently no information available on TGC-specific AIs. The aim of this study is to identify TGC-specific AIs using sera obtained from mice with EAO induced by TGC alone.

## Results

### Explanation of the experimental design

We induced EAO by immunization using viable syngeneic TGC without using any adjuvant. B cell infiltration and IgG deposit were detected by immunohistochemistry in the testis of EAO. IgG titers in TGC-induced EAO mice were extremely high as detected by enzyme-linked immunosorbent assay (ELISA). Then, we identified TGC-specific AIs by TGC liquid chromatography–tandem mass spectrometry (MS) analysis, followed by two-dimensional gel electrophoresis (2D), which showed serum IgG from EAO mice reaction. The expression pattern of identified TGC-specific AIs was analyzed by the Real time PCR. The recombinant proteins of identified 10 (except unnamed protein) TGC-specific AIs were created by using human embryonic kidney 293 (HEK293) cells and these antigencities were reconfirmed by Western blot using EAO serum reaction.

### Histopathological changes in TGC-immunized EAO mice

**Figure 1:**
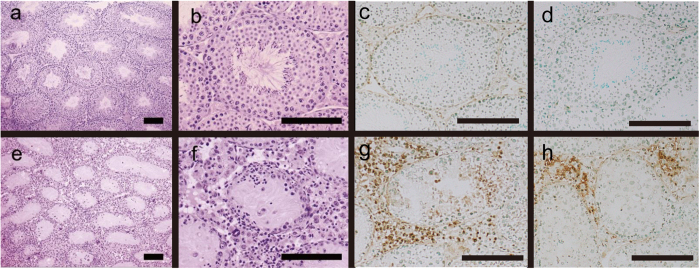
HE staining and B220 and IgG deposit detection on testicular sections from control (**a–d**) and TGC-induced EAO (**e–h**) mice. Testes histological sections from control (**a**,**b**) and TGC-induced EAO (**e**,**f**) mice were stained with hematoxylin and eosin. Additional tissue sections were incubated with specific antibodies to detect B220 (**c**,**g**) and IgG deposits (**d**,**h**) in testes from control (**c**,**d**) and EAO (**g**,**h**) mice. The presence of infiltrating B-cells (**g**) and IgG deposits (**h**) with disrupted spermatogenesis was observed in the testes from TGC-induced EAO mice. Brown spots indicate positive cells (**g**,**h**). Scale bar: 150 μm (**a**–**f**).

No lymphocyte infiltration was observed in any of the control mice testes (Fig. 1a–c). Conversely, extensive lymphocytic infiltration with spermatogenic disturbances was observed in all TGC-immunized EAO mice testes (Fig. 1e–g). Numerous lymphocytes surrounded the peripheral seminiferous tubules, resulting in aspermatogenesis (Fig. 1e–g). No. inflammation was observed in the epididymis of the TGC-immunized EAO mice (data not shown). Immunohistochemical analysis revealed that a portion of the lymphocytes in the interstitium and in the seminiferous tubules were B220-positive cells (Fig. 1g). Moreover, deposits of immunoglobulin (Ig) G were detected a portion of the lymphocytes that accumulated in the interstitium (Fig. 1h). No deposits of IgG and B220 were detected in any of the negative controls (Fig. 1c,d).

### ELISA of TGC autoantibodies in TGC-immunized EAO mice

**Figure 2:**
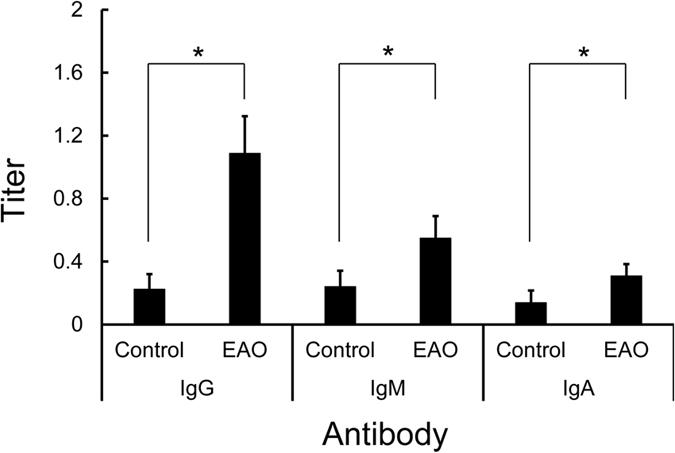
Anti-TGC antibody (IgG, IgA, and IgM) titers in the sera from control and TGC-induced EAO mice. The black bars show the titers from EAO and control mice, respectively. The mean ± SD of five mice per group is presented for each time point. *Significantly different from the titer at controls, p < 0.05.

ELISA analysis revealed that levels of TGC-reactive IgG, IgA, and IgM significantly increased substantially in TGC-induced EAO mice compared with those in control mice (Fig. 2). In particular, IgG titers in TGC-induced EAO mice were extremely high.

### Identification of AI using 2D in TGC-immunized EAO mice

**Figure 3:**
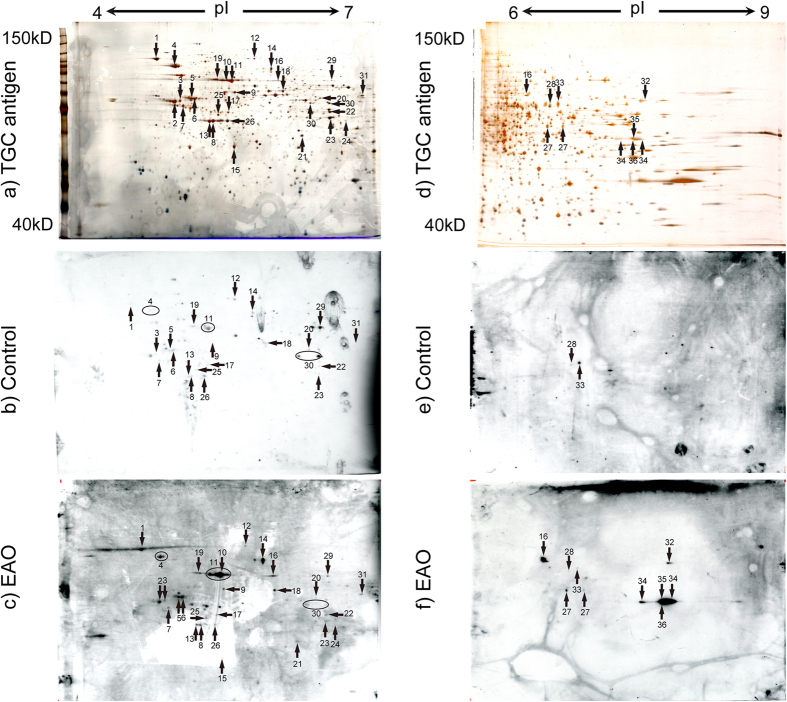
Identification of AIs in TGC proteins by pI4–7 (**a**–**c**) and pI6–9 (**d**–**f**) 2D gel electrophoresis. Numbered proteins were identified on the corresponding silver-stained 2D gel with testicular proteins (**a**,**d**), immunoblotting with control serum (**b**,**e**), and immunoblotting with representative EAO serum (**c**,**f**). The numbered spots indicated proteins that were identified by MS, Tubb2c (spot no. 2), Atp6v1a (spot no. 10), Pdhb (spot no. 15), Hsc70t (spot no. 16), Fbp1 (spot no. 21), Lrrc34 (spot no. 24), Dnpep (spot no. 27), Gapdhs (spot no. 32), Pdha2 (spot no. 34), Dazap1 (spot no. 35), unnamed protein product (spot no. 36) were detected as protein relevant to EAO among the spots. The apparent molecular masses and pIs of the autoreactive proteins were determined by matching them with standard proteins with known pIs and molecular masses.

Because IgG titers in TGC-induced EAO mice were higher than those of IgA and IgM, IgG was used as a secondary antibody for detecting TGC-specific AIs. Spot numbers were only assigned to spots identified by mass spectrometry (MS) using TGC autoantibody as a primary antibody (Table S1). All spots from EAO and control were detected by MS. However, because the spots in which multiple proteins are mixed together, they cannot be identified by MS, thus spot number was not attached. All proteins that visualized on a silver-stained gel were excised and processed for protein identification using MS (Fig. 3a,d). Altogether, isoelectric point (pI) 4–7 set of 29 protein spots (Fig. 3c and Table S1) and pI 6–9 set of 10 proteins spots (Fig. 3f and Table S1) were identified that reacted with EAO serum sample, whereas pI 4–7 set of 23 protein spots (Fig. 3b and Table S1) and pI 6–9 set of two proteins spots (Fig. 3e and Table S1) were identified that reacted with control sera. Some spots (spot no. 1, 3–9, 11–14, 17–20, 22–23, 25–26, 28–31, and 33) overlapped between the EAO and control serum. Other spots (spot no. 2, 10, 15, 16, 21, 24, 27, 32, and 34–36) in EAO serum were not identified in control sera. In effect, we have identified the spots which reacted only with the serum IgG of EAO as TGC-specific AIs. These spots were summarized in Table 1.

### Comparison of AIs mRNA expression between the testes and various other organs by real-time RT-PCR analysis

**Figure 4:**
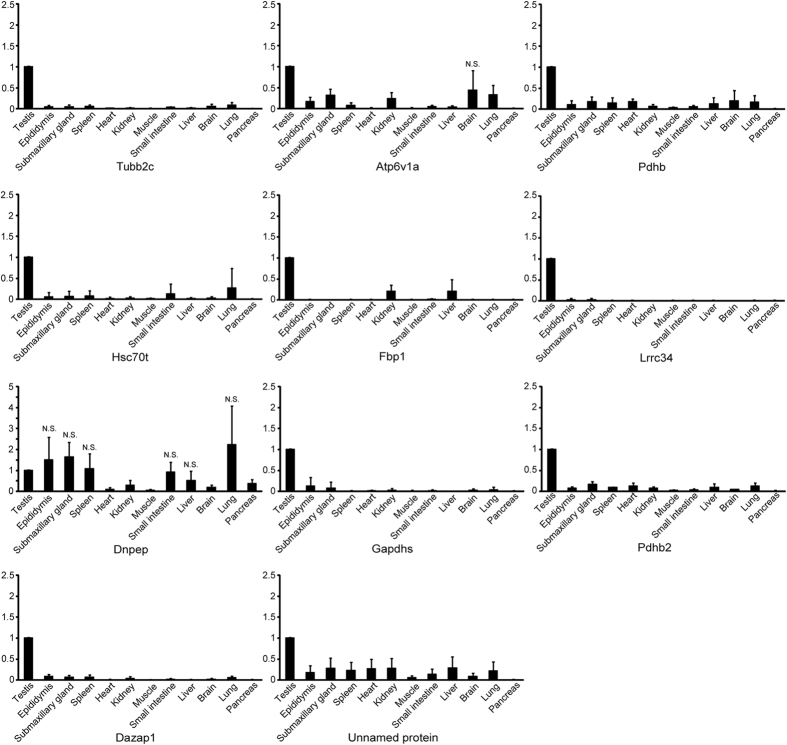
AIs mRNA expression by real-time RT-PCR analysis in various organs. Relative intensity was calculated, after which the expression in the controls (testis) for each point was normalized to 1. Each bar represents the mean ± SD. Significantly different from the vehicle control, p < 0.05. N.S. represents the non–significant values compared to the testis. The remainders with no N.S. were significantly different compared to the testis (p < 0.05).

Figure 4 the mRNA expressions of Tubb2c, Pdhb, Hsc70t, Fbp1, Lrrc34, Gapdhs, Pdha2, Dazap1 and the unnamed protein in testes were significantly higher those in the other organs. Atp6v1a mRNA expression in the testis was significantly higher different between the testis and various other organs except for the brain (p = 0.11). Dnpep mRNA expressions in testes were not significantly different from the ones in epididymis (p = 0.57), submaxillary gland (p = 0.33), spleen (p = 0.88), small intestine (p = 0.82), liver (p = 0.28), lung (p = 0.44), while it was significantly higher than heart, pancreas, kidney, muscle, and brain.

### Comparison of AIs mRNA expression between 2-week- and 8-week-old testes

**Figure 5:**
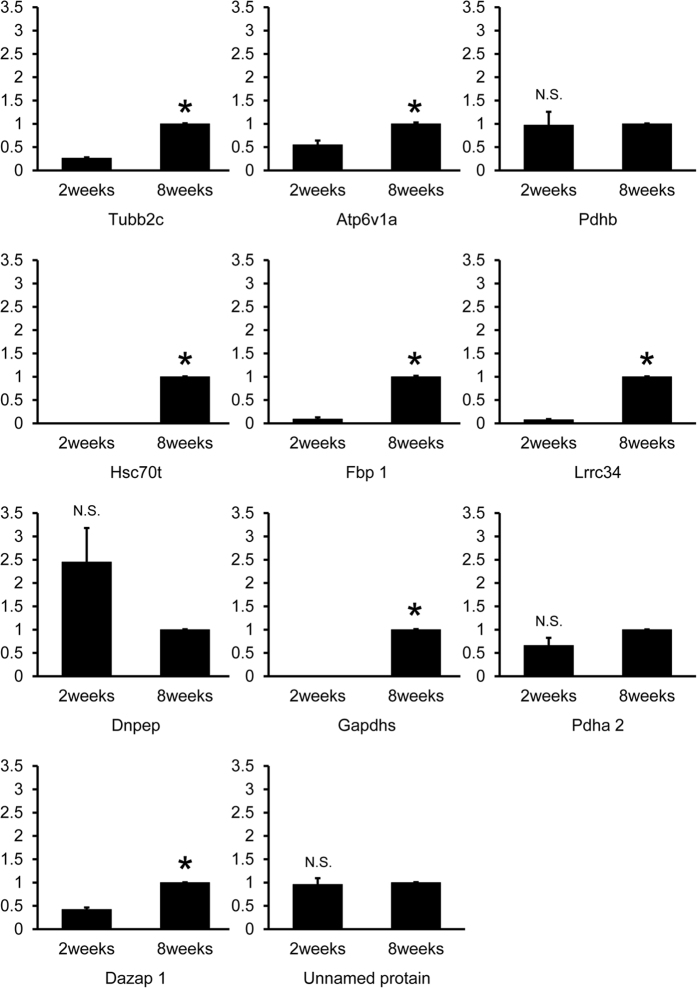
AIs mRNA expression at 2 weeks and 8 weeks of age by real-time RT-PCR analysis. Relative intensity was calculated, after which the expression in the controls (8 weeks) for each point was normalized to 1. Each bar represents the mean ± SD. Significantly different from the vehicle control, p < 0.05. N.S. represents the non–significant values. The asterisks indicate significant differences between 2 and 8 weeks (p < 0.05).

Figure 5 the mRNA expressions of Tubb2c, Atp6v1a, Hsc70t, Fbp1, Lrrc34, Gapdhs and Dazap1 in testes of 8 week old mice were significantly higher than those of 2 week old mice. However, Pdhb (p = 0.88), Pdha2 (p = 0.071), Dnpep (p = 0.075), and the unnamed protein (p = 0.66) showed no such significant difference.

### Comparison of mRNA expressions of AIs between TGC and epididymal spermatozoa

**Figure 6:**
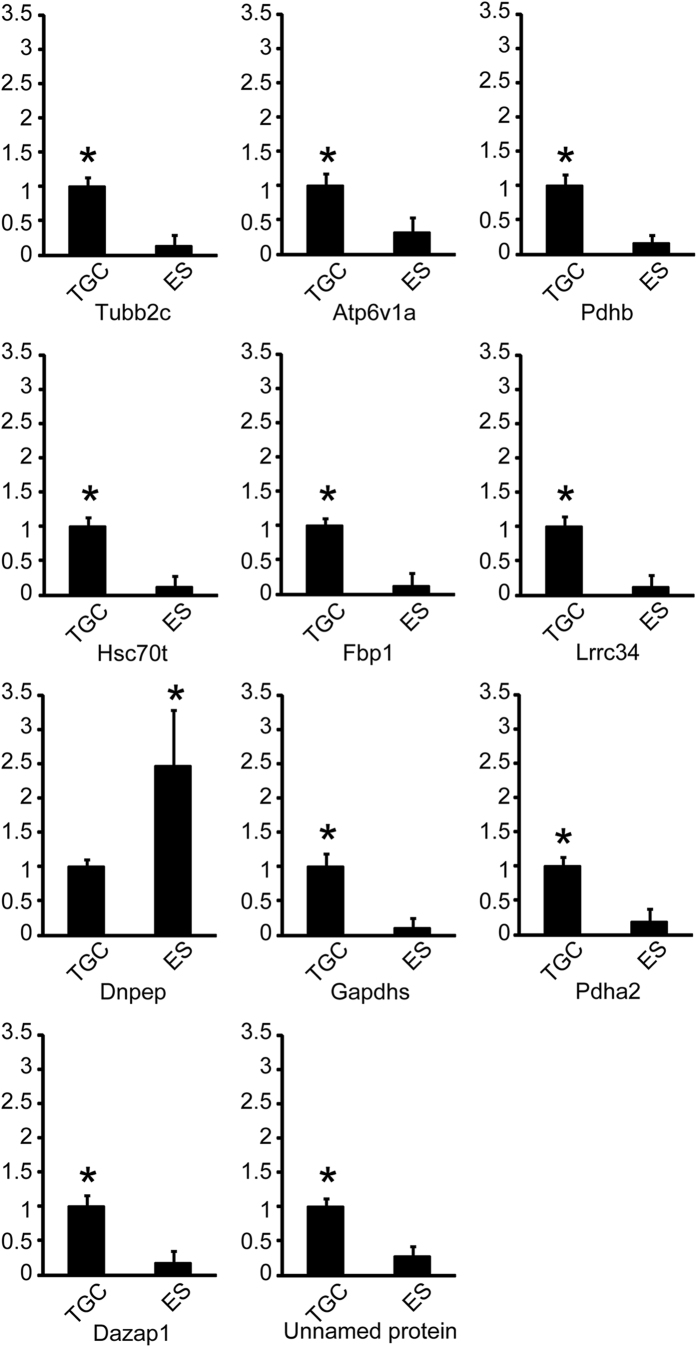
AIs mRNA expression in TGC and ES by real-time RT-PCR analysis. Relative intensity was calculated, after which the expression in the controls (TGC) for each point was normalized to 1. Each bar represents the mean ± SD. The asterisks indicate significant differences between TGC and ES (p < 0.05).

The mRNA expressions of all AIs were significantly different between TGC and epididymal spermatozoa (ES). The mRNA expressions of Tubb2c, Atp6v1a, Pdhb, Hsc70t, Fbp1, Lrrc34, Gapdhs, Pdha2, Dazap1, and the unnamed protein in TGC were significantly higher those in ES. On the contrary, Dnpep in TGC was significantly lower than that in ES (Fig. 6). We decided to include Tubb2c, Atp6v1a, Hsc70t, Fbp1, Lrrc34, Gapdhs, and Dazap1 from the real-time PCR as candidates for TGC-specific AIs.

### Reconfirmation of identified TGC-specific AIs

**Figure 7:**
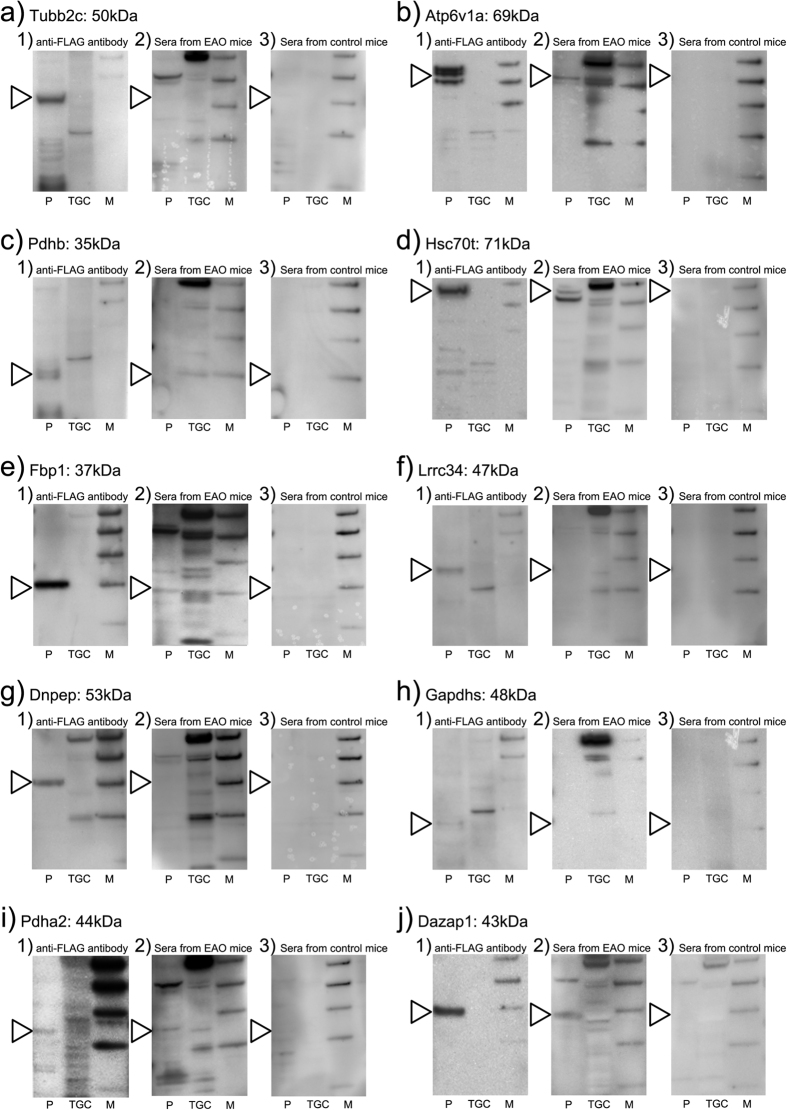
Detection of TGC-specific AIs antibody in EAO serum by Western blot analysis. Tubb2c (**a**) Atp6v1a (**b**) Pdhb (**c**) Hsc70t (**d**) Fbp1 (**e**) Lrrc34 (**f**) Dnpep (**g**) Gapdhs (**h**) Pdha2 (**i**) and Dazap1 (**j**) were transiently expressed in HEK293 cells. The expressed TGC-specific AI proteins by HEK293 (p) and testicular germ cell (TGC) were detected by Western blotting using anti-FLAG antibody (1), EAO sera (2) and control sera (3).

Offered commercially plasmid vectors (Tubb2c, Atp6v1a, Pdhb, Hsc70t, Fbp1, Lrrc34, Dnpep, Gapdhs, Pdha2 and Dazap1) were transfected into human embryonic kidney 293 (HEK293) cells. Expression by HEK293 of TGC-specific AI proteins has been confirmed by FLAG antibody (Fig. 7-1). EAO sera (Fig. 7-2) reacted to various TGC proteins, but control sera did not (Fig. 7-3). Presences of Hsc70t (Fig. 7d), Pdha2 (Fig. 7i) and Dazap1 (Fig. 7j) antibody in EAO serum but not in control serum were determined by Western blot. Presences of Tubb2c (Fig. 7a), Atp6v1a (Fig. 7b), Pdhb (Fig. 7c), Fbp1 (Fig. 7e), Lrrc34 (Fig. 7f), Dnpep (Fig. 7g) and Gapdhs (Fig. 7h) antibody in EAO serum and control serum were not determined by Western blot (Fig. 7).

## Discussion

### Novelty of identified TGC-specific AIs

This study identified 11 AIs using serum autoantibodies from TGC-induced EAO mice. AIs related to testicular autoimmunity have been previously identified using various methods. Primakoff *et al*.^38^ and Tung *et al*.^39^ identified that immunization of male guinea pigs with the sperm surface protein sperm adhesion molecule 1 (SPAM1, PH-20) reproducibly resulted in infertility. They demonstrated that EAO could be induced in guinea pigs immunized with PH-20 + CFA, but they could not induce EAO by the same method in mice^38^,^39^. Recently, Fijak *et al*. reported that disulphide isomerase endoplasmic reticulum 60, heat-shock 70 kDa protein 5, heterogeneous nuclear ribonucleoprotein H1, and sperm outer dense fiber major protein 2 are AIs related to rat EAO in a TH + CFA + BP-induced EAO model^36^. The mice injected with these proteins in CFA had induced EAO at a rate of 25%. PH-20, disulphide isomerase endoplasmic reticulum 60, heat-shock 70 kDa protein 5, and sperm outer dense fiber major protein 2 were expressed in the testis and epididymis in humans and mice^40^,^41^,^42^,^43^. Heterogeneous nuclear ribonucleoprotein H1 was expressed at testicular somatic cells, including Sertoli and Leydig cells, at 0 and 1 week old in mice^36^. Additional expression was observed in spermatocytes and spermatids at 12 weeks in humans and mice^44^. Moreover, following mRNA analysis, it was found that sperm outer dense fiber major protein 2 is expressed in the ovary and uterus^45^, and heat-shock 70 kDa protein 5 is expressed in the lung, pancreas islet, and kidney^46^. Ten of our 11 detected proteins detected are novel AIs that have not been reported.

### TGC-specific AIs details

#### Relevance to EAO with respect to molecular weight and age of the identified TGC-specific AIs

We previously investigated AIs relevant to TGC-induced EAO through reacting individual immune serum samples with testes from normal mice of various ages using immunoblotting^47^. The results showed that the sera obtained from mice with TGC-induced EAO lesions specifically defined testicular antigens with molecular weights of 15 kDa, 40 kDa, 75 kDa, and >200 kDa from 4-week-old mice^47^. In addition to these, bands of 24–35 kDa and 60–75 kDa were detected in testicular proteins at 8 weeks of age^47^. Moreover, we incubated immunoblots with EAO serum from various weeks after TGC immunization^32^. At 0 week of age, a band corresponding to approximately 40 kDa was detected in TGC proteins, indicating the presence of natural autoantibodies against TGC. At 4 weeks of age, in addition to this band, a band of approximately 42 kDa was detected in TGC proteins. At 8 weeks of age, three TGC protein bands (approximately 20, 28, and 60 kDa) were also detected^32^. Considering the EAO-inducing factors and expression levels in testis at 2 weeks and 8 weeks, the EAO-related proteins were 24–35, 42, and 60–75 kDa. In this study, we identified 11 AIs recognized by serum antibodies from TGC-induced EAO in mice extracted Tubb2c (50 kDa), Atp6v1a (69 kDa), Hsc70t (71 kDa), Fbp1 (37 kDa), Lrrc34 (47 kDa), Gapdhs (48 kDa), Pdha2 (44 kDa), Dazap1 (43 kDa), and the unnamed protein product (55 kDa) from mRNA expression analysis at 2 weeks and 8 weeks of age. Therefore, Atp6v1a, Hsc70t, Fbp1 Lrrc34, Gapdhs, and Dazap1 can reasonably be categorized as EAO-related proteins.

#### Localization of the identified TGC-specific AIs and relevance to EAO

Information from the literature on the 11 identified AIs is presented in Table 2^48^,^49^,^50^,^51^,^52^,^53^,^54^,^55^,^56^,^57^,^58^,^59^,^60^,^61^,^62^,^63^. Tubb2C, Fbp1, Pdhb, and Gapdhs are all expressed in the sperm tail. Pdha2 is also expressed in haploid germ cells, diploid germ cells, and Sertoli cells. Hsc70t, Gapdhs and Fbp1 are expressed in spermatids. Lrrc34 and Dazap1 are expressed in spermatocytes and spermatids. Thus, Lrrc34, Hsc70t and Dazap1 appear candidate EAO-related AIs based on the present study results and previous literatures. Further, information of TGC-specific AIs by using immunohistochemistry may contribute to elucidate the pathological mechanism of EAO.

#### Focus of candidate TGC-specific AIs

We have examined the candidates for TGC-specific AIs from the literature and our experiment. We decided to include Tubb2c, Atp6v1a, Hsc70t, Fbp1, Lrrc34, Gapdhs and Dazap1 as the candidates for TGC-specific AIs from the results of real-time PCR (tissue specificity, age, cell type) in this study. In addition, we selected to include Atp6v1a, Hsc70t, Fbp1 Lrrc34, Gapdhs, and Dazap1 based on molecular weight and age from the literature as potential candidate TGC-specific AIs. Furthermore, we have decided to include Lrrc34, Hsc70t and Dazap1 from testicular localizations, as described in the literature as candidate TGC-specific AIs. Finally, the recombinant proteins of identified 10 (except unnamed protein) TGC-specific AIs were created by using human embryonic kidney 293 (HEK293) cells and these antigencities were reconfirmed by Western blot using EAO serum reaction. The results indicated Atp6v1a, Hsc70t, Fbp1 and Dazap1 were candidates for TGC-specific AIs.

### Usefulness of the information of identified TGC-specific AIs

#### Information on TGC-specific AIs identified in germ cell developmental biology

In male germ cells, many marked morphological changes occur during spermatogenesis, particularly in haploid spermatids after meiotic division^64^. In various mammals, male germ cell differentiations proceed actively and continuously in the testis after puberty, and sperms are produced throughout adulthood^64^. Mice require 1 month for completion of spermatogonial stem cell proliferation and differentiation, meiosis, generation of haploid germ cells, and morphogenesis of the developing testicular spermatozoa in seminiferous tubules^64^. After meiotic division (during the process of haploid germ cell differentiation, or spermiogenesis), the rounded spermatids undergo marked morphological changes to become testicular spermatozoa: the nucleus assumes a compact shape, the mitochondria are rearranged, the flagellum forms, and the acrosome is generated. During this period of differentiation, which occurs within 5–6 weeks in humans^65^,^66^ and 2–3 weeks in mice^67^, haploid germ cells do not divide but morphogenesis occurs, indicating that some regulatory mechanism arrests the cell cycle. Searching for functional changes in genes and gene products involved in male infertility would increase our understanding of the causes of this condition and perhaps lead to new treatments for some cases. The simplest strategy for elucidating the mechanism of spermatogenesis is to identify and characterize differentiation-specific molecules and their associated genes in germ cells. In this study, the mRNA expressions of 10 AIs, excluding Dnpep, were significantly higher in mice at 8 weeks of age compared with those at 2 weeks and in TGC compared with epididymal spermatozoa (ES). Thus, these proteins may be involved in the development of testicular spermatozoa.

#### Information on identified TGC-specific AIs as cancer/testis antigens (CTs)

CTs, also known as cancer germline antigens, refer to a growing list of antigens that were initially discovered in the 1980 s–1990 s, which are specifically expressed in various tumor types^68^,^69^. At present, more than 70 CTs are present families encompassing more than 140 individual members with largely unknown functions^69^,^70^. Because each cancer type is associated with multiple highly expressed CTs, an effective vaccine may require the presence of multiple CTs. CTs are absent in normal humans and rodent somatic cells and are expressed only in male TGC^34^,^71^,^72^,^73^,^74^,^75^. In this study, the mRNA expressions of seven TGC-specific AIs (including Tubb2c, Atp6v1a, Hsc70t, Fbp1, Lrrc34, Gapdhs and Dazap1) were significantly higher in testis, particularly in male germ cells, compared with that in other organs in 8-week-old mice than in 2-week-old mice. Generally, CTs can be grouped into two classes based on their chromosomal location: CTs-X are located on the X chromosome and non-X CTs are located on the autosomes. Most CTs-X are unique to primates and constitute several subfamilies of homologous genes, organized in discrete clusters along the X chromosome^76^. As a result of their restricted expression in an immune-privileged organ, both CTs groups represent attractive immunotherapy targets^34^. Antigenic CTs-derived peptides that are presented to the immune system with different human leukocyte antigen allospecificities elicit both humoral and cellular immune responses. Spontaneous humoral and cell-mediated immune responses have been demonstrated for several CTs and patients with good antibody titers often present with a better prognosis^71^. TGC-induced EAO involves both cellular and humoral immune responses to the autoantigen-containing AIs. Therefore, we expect that cellular and humoral immunoreactions to AIs can be identified in TGC-induced EAO. Although emerging evidence has clarified the functions of a few CTs-X in cancer, the majority remains poorly understood. In contrast, non-X CTs are fairly well conserved throughout evolution, with established roles in processes such as transformation^76^, chromatin remodeling^34^, transcriptional regulation, and cell signaling^72^. Thus, non-X CTs are particularly promising targets for the development of small-molecule therapeutics^77^. In fact, we examined whether the candidate TGC-specific AIs are upregulated in cancer cells or tumors from previous reports (Table 3). These studies suggested that Tubb2c, Pdhb, Hsc70t, Fbp1 and Gapdhs are upregulated in some cancer cells or tumors (Table 3). A list of CTs has been first manually compiled from the literature (http://www.cta.lncc.br) and the resultant *CT Database* contains 204 genes^78^. All TGC-specific AIs identified in this study are encoded on an autosomal chromosome.

## Conclusion

Using serum autoantibodies from mice immunized with only syngeneic TGCs alone, we have identified 11 proteins as respective testicular AIs. Real-time RT-PCR analysis showed that the mRNA expressions of seven TGC-specific AIs (Tubb2c, Atp6v1a, Hsc70t, Fbp1, Lrrc34, Gapdhs, Dazap1) were significantly higher in only mature testis compared to other organs. Three TGC-specific AIs (Hsc70t, Lrrc34, Dazap1) were shown as EAO-related proteins in previous reports and the present study also. Seven TGC-specific AIs (including Tubb2c, Atp6v1a, Hsc70t, Fbp1, Lrrc34, Gapdhs and Dazap1) show human homology. Finally, the recombinant proteins of identified 10 (except unnamed protein) TGC-specific AIs were created by using human embryonic kidney 293 (HEK293) cells and these antigencities were reconfirmed by Western blot using EAO serum reaction. These results indicated Atp6v1a, Hsc70t, Fbp1 and Dazap1 were candidates for TGC-specific AIs. Information of AIs have also received considerable attention because of their role as cancer/testis antigens (CTs). Identification of these AIs will facilitate new approaches for understanding infertility and cancer pathogenesis and may provide a basis for the development of novel therapies. In the future, we are planning to generate some recombinant proteins of TGC-specific AIs.

## Materials and Methods

### Animals

A/J mice (8-week-old) were purchased from SLC (Shizuoka, Japan) and maintained in the Laboratory Animal Center of Tokyo Medical University for 2 weeks before use. The mice were maintained at 22 °C–24 °C and 50–60% relative humidity with a 12-h light–dark cycle. After approval by the Tokyo Medical University Animal Committee (S-23041, S-24018), all animal experiments were performed in accordance with the guidelines of the National Institute of Health.

### Preparation of TGC

Testes were excised from 10- and 2-week-old mice (n = 10 each), minced with scissors in cold Hanks’ balanced salt solution (HBSS), and passed through a stainless steel mesh. TGCs were harvested by centrifugation at 400 G for 15 min, washed three times in cold Hanks’ balanced salt solution, and then adjusted to a concentration of 1 × 10^7^ TGC/200 μl/mouse after determining cell viability by trypan blue dye exclusion. The TGC suspension contained more than 99% germ cells at various stages of spermatogenesis; the remaining <1% consisted of Sertoli and interstitial cells^21^. The ratio of prepared cells was determined by visual microscopic inspection using a hemocytometer. In addition, prepared cells have been confirmed by immunohistochemical method and real-time RT-PCR (Figure S1).

### Induction of EAO by TGC

At 10 weeks of age, male mice were subcutaneously injected with 1 × 10^7^ TGC/mouse once on day 0 and again on day 14 (i.e., at a 2-week interval) for induction of active EAO. Male mice (aging 10 weeks) injected with HBSS alone were used as controls. At 120 days after the first immunization, the mice were deeply anesthetized with pentobarbital (65 mg/kg body weight) and their testes were removed. Histopathological samples were taken from the right testes, and immunohistochemical samples were collected from the left testes of the mice (n = 10 for both the TGC-immunized mice and control mice). Blood samples were collected from the mice by cardiac puncture (n = 10 for each group).

### Examination of inflammatory severity of EAO by hematoxylin and eosin staining

Isolated right testes obtained from EAO-affected mice and control mice were fixed with Bouin’s solution and then embedded in plastic (Technovit 7100; Kulzer & Co., Wehrheim, Germany) without cutting the organs to prevent artificial damage to the testicular tissue. Sections (3–4-μm thick) were obtained at 15–20-μm intervals, stained with Gill No. 3 hematoxylin and 2% eosin Y (HE stained), and observed with a light microscope (BX51; Olympus, Shinjuku, Japan).

### Examination of inflammatory factor by B220 and IgG deposit stain

Isolated left testes obtained from EAO-affected mice and control mice were placed in OCT compound (Miles Laboratories, IL, USA), frozen in liquid nitrogen, and stored at −80 °C until use. Five-micrometer-thick sections were cut with a cryostat (CM1900; Leica, Wetzlar, Germany) and then fixed in ethanol for 10 min at −20 °C. The sections were then rinsed in phosphate-buffered saline (PBS) and incubated with Block Ace (Yukijirushi, Hokkaido, Japan) for 20 min at room temperature to inactivate endogenous peroxidase activity. After rinsing in PBS, the sections were incubated with a rat anti-mouse B220 (clone: RA3-6B2, ×200; BD Biosciences) monoclonal antibody, followed by incubation with rabbit anti-rat IgG (Vector Labs, CA, USA) at room temperature. Bound antibodies were detected by incubation with horseradish peroxidase (HRP)-conjugated goat anti-mouse IgG (ZyMax, South San Francisco, CA) at room temperature. Immunoreactive cells were visualized using a Vectastain ABC Kit (Vector Labs, CA, USA) with 3,3′-diaminobenzidine (DAB) as the chromogen. Bound HRP was detected using 0.05% DAB and 0.01% H_2_O_2_. Sections processed with rabbit serum instead of the primary antibodies were used as negative controls. The stained sections were counterstained with methylgreen (Vector Laboratories, CA, USA).

### Examination of autoantibody (IgG, IgM, IgA) titer by ELISA

ELlSA was performed using the method described by Hirai *et al*.^32^. For preparation of antigens, TGC obtained from 10-week-old normal mice (n = 3) was homogenized in carbonate–bicarbonate buffer (Sigma-Aldrich, MO, USA). Antigen concentrations were determined using the Bradford method with bovine serum albumin as the standard. Antigen was then added to each well of a microtiter plate (Nunc 96-well plate; Thermo Fisher Scientific, Kanagawa, Japan) and incubated at 37 °C for 30 min. After removing the coating solution, the wells were washed three times with PBS-Tween 20; Then, 200 μl of a rabbit anti-mouse monoclonal glyceraldehyde 3-phosphate dehydrogenase (GAPDH) antibody (housekeeping gene; Bethyl Laboratories, Inc., TX, USA; 1/200 and 1/1000 dilution) or experimental mouse serum samples serially diluted with 1% goat serum in PBS (each sample run in duplicate) were added to microELISA wells and incubated for 2-h at room temperature. The wells were then washed five times with PBS-Tween 20 and incubated for 2-h at RT with 50 μl of HRP-conjugated goat anti-rabbit IgG (Cappel, PA, USA; 1:1,000), anti-GAPDH antibody, or HRP-conjugated goat anti-mouse IgG (Cappel; 1:1,000). A solution of one Alkaline Phosphatase Yellow (pNPP) tablet and one Tris Buffer tablet (Sigma-Aldrich) dissolved in 5 ml of water was used as a soluble substrate to detect alkaline phosphatase activity. After the wells were again washed five times with PBS-Tween 20, 200 μl of freshly prepared pNPP solution was added to each well. Thirty minutes later, the absorbance at 492 nm was determined using a microtiter plate reader (MPR-A4, Toyo Soda, Tokyo, Japan).

### Separation of testicular proteins using two-dimensional gel electrophoresis

Testes from normal 10-week-old mice (n = 3) were lysed by sonication in isoelectric focusing rehydration buffer (7 M urea; 2 M thiourea; 4% CHAPS; 100 mM DTT; 0.2% Bio-Lyte, pH 5–8; 0.01% bromophenol blue; and protease inhibitor). Insoluble material was pelleted (12,000 g, 15 min and 100,000 g, 60 min) and the resulting supernatant was desalted using a 2D Cleanup Kit (GE Healthcare UK Ltd, Buckinghamshire, England). One hundred microgram of protein in a total of 200 μl of rehydration buffer was applied to 24-cm pI gradient strips (pI4–7 and pI6–9; GE Healthcare UK, Buckinghamshire, England) for overnight rehydration. First-dimension isoelectric focusing was preformed on a GE Multiphor II System (GE Healthcare UK). After focusing, strips were equilibrated with equilibration buffer (6 M urea; 50 mM Tris-HCl, pH 8.0; 2% SDS; 20% glycerol; and 2% w/v DTT) for 15 min. The strips were further equilibrated with equilibration buffer II (6 M urea; 50 mM Tris-HCl, pH 8.0; 2% SDS; 20% glycerol; and 2.5% w/v iodoacetamide) for 15 min and then directly applied to a 7.5% isocratic SDS-polyacrylamide gel for the second dimension separation. The resulting gel was then transferred to a membrane for Western blot or stained using a silver stain kit (Wako Pure Chemical Industries, Osaka, Japan).

### AI protein identification by Western blot and MS

In mice immunized with isolated TGC alone, only EAO is inducible without epididymo-vasitis^21^. This EAO model has been found to involve Th1-cell-dependent autoimmunity; however, B-cell lineages were also related to the development of EAO in the present study. Several IgG immune deposits are present in EAO lesions. Various immunoglobulins (IgG, IgM, and IgA) against TGC were detected in TGC-induced EAO mice sera in the present study. TGC-induced EAO is unique as serum autoantibodies are only produced against the acrosomes of ES and round spermatids^25^,^26^,^32^. Previously, we had observed that there were some molecular differences between TGC and ES antigens that reacted with autoantibodies using Western blot^32^. Considering that epididymitis does not occur in TGC-induced EAO, the AI analysis was performed using TGC rather than ES.

After gel electrophoresis, proteins were transferred onto a nitrocellulose membrane. Membranes were blocked in 4% skimmed milk in TBST for 1 h, followed by serial incubation with EAO and normal serum samples (1:50) 4 °C overnight, a biotinylated goat anti-mouse whole IgG antibody (Amersham Biosciences, Freiburg, Germany; 1:10000), and finally, a streptavidin–HRP conjugate for 30 min (Amersham Biosciences). Bound antibodies were visualized using the ECL Plus Detection Reagent (Amersham Biosciences). Spots corresponding to the Western blot membrane were cut from the silver stained 2D gel for identification by MS. Peptidase protein digestion was performed as follows: A piece of a gel spot stained with silver was placed in a sampling tube, dried with a vacuum concentrator, rehydrated with a trypsin solution (10 μm/ml in 50 mM ammonium bicarbonate) and then placed in a small volume of 50 mM ammonium bicarbonate. After the incubation at 37 °C under vigorous shaking for 12–16 h, the supernatant solution was removed and stored for further use. The gel piece was successively extracted once with 50 μl and again with 25 μl of 5% trifluoroacetic acid/50% acetonitrile with shaking for 30 min each time. The supernatant and extracts were mixed and concentrated to a volume of 5–10 μl.

### Examination of expression of TGC-specific AIs gene (tissue specificity, age, cell type) by gene expression analysis

Testes, epididymis, submaxillary gland, spleen, heart, kidney, muscle, small intestine, liver, brain, lung, and pancreas from normal control A/J mice were evaluated at 8 weeks of age (n = 4) for expression analysis in the various organs. Testes from normal A/J mice were evaluated at 2 weeks of age (n = 4) for analysis of expression that was related to spermatogenesis. TGC and ES were excised from 10-week-old mice (n = 4), minced with scissors in cold HBSS, and passed through a stainless steel mesh. TGC and ES were harvested by centrifugation at 400 g for 15 min and washed three times in cold HBSS.

For analysis, total RNA was isolated from the entire above organs and cells using the TRIzol RNA Extraction Kit (Invitrogen, CA, USA), according to the manufacturer’s instructions, and the RNA pellets were dissolved in 10 ml of RNase-free distilled water. Total RNA was measured at 260/280 nm using a UV spectrophotometer and was stored at −80 °C prior to use. cDNAs were prepared from 10 μg of total RNA in a 100 μl reaction mixture using random primers according to a standard protocol (high capacity cDNA archive kit; PE Applied Biosystems, Foster City, CA, USA). The PCR reactions were conducted using an iCycler thermal cycler (Bio-Rad, Hercules, CA, USA), and the mixtures were stored at −80 °C until analysis. Real-time RT-PCR was performed using 3 ng of cDNA and the validated SYBR Green gene expression assay in combination with the SYBR Premix Ex Taq II (TaKaRa, Bio Inc., Ohtsu, Japan) for measuring Tubb2c, Atp6v1a, Pdhb, Hsc70t, Fbp1, Lrrc34, Tyrp1, Gapdhs, Pdha2, Dazap1, Fh1, and GAPDH. All of the primers used in this analysis are listed in Table 4. Quantitative real-time PCR was performed in duplicate in a thermal cycler dice real time system TP800 (TaKaRa). The data were analyzed using thermal cycler dice real time system software (TaKaRa), and the comparative C_t_ method (2∆∆C_t_) was used to quantify gene expression levels. Real-time RT-PCR data were standardized to GAPDH, which was used as an internal control. To confirm the specific amplification of the target genes, each gene product was further separated on a 1.5% agarose gel to detect any single bands at the theoretical product sizes, and the dissociation curves were analyzed to detect any single peaks.

### Examination of expression of TGC-specific AIs gene by Human embryonic kidney 293 (HEK293) cells

Plasmid vectors (pCMV6) of TGC-specific AIs and Native were purchased from Origene (Table S2). All proteins were expressed in a suspension-adapted HEK293 cell line (ExPi293F, Thermo Fisher). Cells, growing in deep 6-well blocks, were transfected in triplicate at a density of 1.0 × 107/ml using ExpiFectamine™ 293 according to the manufacturer’s instructions. Following transfection, the cells were grown at 37 °C by shaking in 5% CO_2_, for 48 hours. The conditioned media, containing secreted protein, were harvested by centrifugation at 1000 rpm for 5 minutes. The clarified media from each of the three replicates for each expression vector were pooled and stored at −20 °C. Each transfection was performed in duplicate, on separate days, and the conditioned media was analyzed separately by Western blot analysis.

### Detection of TGC-specific AIs in EAO serum by Western blot analysis

The expression of TGC-specific AI proteins by HEK293 and testicular germ cells were evaluated by Western blot analysis for detection of the presence of TGC-specific AIs antibody in EAO serum. The concentration of protein was measured with a Protein Quantification Kit-Rapit (Dojindo Molecular Technologies, Kumamoto, Japan). Samples were mixed with LDS Sample Buffer (Thermo Scientific, Rockford, IL, USA) and an equal amount of protein per lane was run on a 4–12% SDS-PAGE and transferred by iBlot (Thermo Scientific, Rockford, IL, USA). Blots were incubated with anti-FLAG antibody (Sigma-Aldrich, MO, USA) at a dilution of 1:1000, EAO serum at a dilution of 1:50, control serum (10 week mice) at a dilution of 1:50 at 4 °C overnight, followed by incubation with horseradish peroxidase-conjugated sheep anti-mouse IgG second antibody (GE Healthcare, Little Chalfont, UK) at a dilution of 1:10000 for 2 h at room temperature. The proteins were visualized by chemiluminescence using an ECL Prime western blotting detection kit (GE Healthcare, Little Chalfont, UK) according to the manufacturer’s instructions. Detection was performed with a ImageQuant 350 (GE Healthcare, Little Chalfont, UK).

### Data analyses

Data are expressed as the mean ± standard devtion (SD). ANOVA and the Tukey’s multiple comparison tests were employed for statistical analysis. All tests were performed as two-sided test and a p value of <0.05 was accepted as significant.

## Additional Information

**How to cite this article**: Terayama, H. *et al*. Specific autoantigens identified by sera obtained from mice that are immunized with testicular germ cells alone. *Sci. Rep.* **6**, 35599; doi: 10.1038/srep35599 (2016).

## Supplementary Material

Supplementary Information

## Figures and Tables

**Table 1:** TGC-specific AIs identified by Western blot and MS analysis.

Spot no	Name	MW	pI	Other symbols
2	Tubulin, beta 2c1 (Tubb2c)	50239	4.79	4930542G03Rik, MGC:28623, MGC:6713, Tubb4b
10	ATPase, H+ transporting, lysosomal V1 subunit A (Atp6v1a)	68553	5.48	Atp6a1, lysosomal 70kDa, VA68, VPP2
15	Pyruvate dehydrogenase (lipoamide) beta (Pdhb)	35156	5.63	2610103L06Rik
16	Heat shock protein 1-like: Hspa1l (Hsc70t)	70978	5.81	70kDa, Hspa1L, Msh5
21	Fructose bisphosphatase 1 (Fbp1)	37288	6.15	Fbp-2, Fbp3, FBPase brain isoform, FBPase liver
24	Leucine rich repeat containing 34 (Lrrc34)	47118	6.38	1700007J06Rik
27	Aspartyl aminopeptidase isoform b (Dnpep)	52744	6.82	—
32	Glyceraldehyde-3-phosphate dehydrogenase, spermatogenic (Gapdhs)	48096	8.17	Gapds, Gapd-s
34	Pyruvate dehydrogenase E1 alpha 2 (Pdha2)	44183	8.79	Pdhal
35	DAZ-associated protein 1 (Dazap1)	43281	8.88	2410042M16Rik, mPrrp
36	Unnamed protein product (Fumarate hydratase related protein)	54550	9.12	—

**Table 2:** Detailed information on the 11 identified TGC-specific AIs.

Spot no	Name	Function	Reported Species	Detection Method	Localization
2	Tubb2c	cytoskeletal protein	human	electron microscopy	sperm flagellum
10	Atp6v1a	hydrolase in generating and maintaining the acidity of organelles	rat	protein expression (immunohistchemistry)	epididymal epithelial cell
15	Pdhb	dehydrogenase for converting pyruvate to acetyl CoA	hamster	protein expression (immunohistchemistry)	sperm flagellum
16	Hsc70t	heat shock protein	pig, fox, horse, cattle, marmoset, Japanese monkey, human, musk shrew, blue-white dolphin, dunnart	protein expression (immunohistchemistry)	late spermatid (cytoplasm)
21	Fbp1	rate-limiting step enzymes in gluconeogenesis	rat	protein expression (immunohistchemistry)	round and elongating spermatid (cytoplasm and flagellum)
24	Lrrc34	protein in forming solenoid-shaped structures for protein-protein interactions	mouse	mRNA expression (*in situ* hybridization)	late spermatocyte, round spermatid
27	Dnpep	—	—	—	—
32	Gapdhs	oxidoreductase in glycolysis	human, rat, mouse	protein expression (immunohistchemistry)	spermatid, epididymal spermatozoa
34	Pdha2	dehydrogenase for converting pyruvate to acetyl CoA	human	mRNA expression (quantitative RT-PCR)	Sertoli cells, germ cells, ejaculated spermatozoa
35	Dazap1	RNA binding proteins	mouse	protein expression (immunohistchemistry)	late pachytene spermatocyte, round spermatid (nuclei) elongating spermatid (cytoplasm)
36	Unnamed protein product	—	—	—	—

**Table 3:** Upregulation in the candidate of TGC-specific AIs in cancer cells or tumors under the previous reports.

Name of AIs	Materials	Methods	Related cancer or tumor	value of fold	Reference
Tubb2c	Epstein-Barr virus (EBV) + Raji cell line (Endemic Burkitt lymphoma) EBV + NC37 cell line EBV – Ramos cell line (Sporadic Burkitt lymphoma)	Mass spectrometry-based isotope tag for relative and absolute quantitation (iTRAQ)	Burkitt lymphoma	5.2	79
Tubb2c	HEC-1A cell line	Mass spectrometry	Endometrial cancer cells	not shown	80
Tubb2c	Bone marrow cell	Gene expression microarray	Myelodysplastic syndrome	not shown	81
Atp6v1a	—	—	—	—	—
Pdhb	DLD1 cell line	Real-time PCR	Colon carcinoma	1.5	82
Pdhb	Non-side population in 50 CNE-2 single cell	Northern blot	Nasopharyngeal carcinoma	not shown	83
Hsc70t	Hepatocellular carcinoma cell	Gene expression microarray	Hepatocellular carcinoma	1.1	84
Hsc70t	A375 metastatic melanoma cell line	RT2 Human Stress and Toxicity Profiler™ PCR Expression Array	Melanoma	16.9	85
Fbp1	C666, CNE2, S18, S26, SUNE2, 5-8F, 6-10B, SUNE1, CNE1, HNE1, HK1 and HONE1 (NPC cell lines)	Western blot	Nasopharyngeal carcinoma	2.8, 5.6, 3.1, 2.3, 2.9, 1.7, 3, 4.1, 2.9, 3.3, 2.5, 2.9	86
Fbp1	Pancreatic cancer cell lines (Panc-1, Bxpc-3, Aspc-1)	Real-time PCR	Pancreatic cancer	2.5	87
Fbp1	Hepatocellular carcinoma cell MH14 cell line	Immunohistochemistry and Oncomine data analysis (mRNA expression)	Hepatocellular carcinoma (with chronic hepatitis C)	4	88
Fbp1	Glioma cell	Western blot	Glioma	2	89
Fbp1	Lung cancer cell	Mass spectrometry	Lung cancer	2.5	90
Fbp1	CCLP1 human liver cancer cell line	Western blot	Liver cancer	1.72	91
Fbp1	Bladder urothelial carcinoma tissue	Immunohistochemistry	Bladder urothelial carcinoma	not shown	92
Lrrc34	—	—	—	—	—
Dnpep	—	—	—	—	—
Gapdhs	—	—	—	—	—
Pdha2	—	—	—	—	—
Dazap1	—	—	—	—	—
Unnamed protein	—	—	—	—	—

**Table 4:** Real time RT-PCR primers.

Spot no	Target Name	Foreword Primer	Reverse Primer
2	Tubb2c	gctcctcttctacagctgttcc	gctgattacctcccagaacttg
10	Atp6v1a	aacgctgggtattgttcagg	agtgcttgcgctgagctaac
15	Pdhb	gatgaagacaaatcatctcgtgac	tctggcacaaatctcagctc
16	Hsc70t	ctggatcgaaggcgtagaga	ccgatcgccattcctttat
21	Fbp1	tataccccgccaacaagaaa	aagctatggggttgcactca
24	Lrrc34	gggctctaggcgaaggaa	agcttctccatcgactctgg
27	Dnpep	ctgggtggaggtcctcttg	cgacagatgtggattcctagc
32	Gapdhs	ccttgagatcaacacgtaccag	cgcctgtacactccaccac
34	Pdha2	gaggacacgggcaagatg	aatagcttcacgaactgtcaactg
35	Dazap1	gccccagacatgagcaaa	aagtcctgcccgtaacctg
36	Unnamed protein	gcaccccaatgatcatgtta	attgctgtgggaaaggtgtc
