# Supplementary material for: Specific autoantigens identified by sera obtained from mice that are immunized with testicular germ cells alone
Source: Sci Rep. 2016 Oct 18;6:35599. doi: 10.1038/srep35599 (PMC5067510; doi:10.1038/srep35599)
Supplement: Supplementary Information [file srep35599-s1.doc]

Specific autoantigens identified by sera obtained from mice that are immunized with testicular germ cells alone

Hayato Terayama^1,2¶^*, Shuichi Hirai^2,3¶^, Munekazu Naito^2,3^, Ning Qu^2^, Chiaki Katagiri^4^, Kenta Nagahori^2^, Shogo Hayashi^2^, Hiraku Sasaki^5^, Shota Moriya^6^, Masaki Hiramoto^6^, Keisuke Miyazawa^6^, Naoyuki Hatayama^2,3^, Zhong-Lian Li^2^, Kou Sakabe^1^, Masayuki Matsushita^4^, Masahiro Itoh^2^

^1^ Department of Anatomy, Division of Basic Medical Science, Tokai University School of Medicine, Kanagawa, Japan

^2^ Department of Anatomy, Tokyo Medical University, Tokyo, Japan

^3^ Department of Anatomy, Aichi Medical University, Aichi, Japan

^4^ Department of Molecular and Cellular Physiology, Graduate School of Medicine, University of the Ryukyus, Okinawa, Japan

^5^ Department of Health Science, School of Health and Sports Science, Juntendo University, Chiba, Japan

^6^ Department of Biochemistry, Tokyo Medical University, Tokyo, Japan.

^¶^These authors contributed equally to this work.

Supplementary Table S1

List of all spots reacted by control and EAO serum.

| **Spot no** | **Name** | **MW** | **pI** | **Normal** |
| --- | --- | --- | --- | --- |
| **1** | **Tumor rejection antigen gp96 (Hsp90b1)** | **92703** | **4.74** | **+** |
| **2** | **Tubulin, beta 2c1 (Tubb2c)** | **50239** | **4.79** | **-** |
| **3** | **Tubulin, beta 3 (Tubb3)** | **50842** | **4.82** | **+** |
| **4** | **Heat shock protein 1, alpha (Hsp90aa1)** | **85134** | **4.93** | **+** |
| **5** | **Tubulin, alpha 1b (Tuba1b)** | **50804** | **4.94** | **+** |
| **6** | **Tubulin, alpha 1a (Tuba1a)** | **50612** | **4.97** | **+** |
| **7** | **ATP synthase, H+ transporting mitochondrial F1 complex, beta subunit (Atp5b)** | **56265** | **5.19** | **+** |
| **8** | **Unnamed protein product (Actin related protein)** | **42066** | **5.3** | **+** |
| **9** | **Chaperonin subunit 8 (Theta)** | **60088** | **5.44** | **+** |
| **10** | **ATPase, H+ transporting, lysosomal V1 subunit A (Atp6v1a)** | **68553** | **5.48** | **-** |
| **11** | **Heat shock protein 2 (Hspa2)** | **69884** | **5.51** | **+** |
| **12** | **Heat shock protein 4 like (Hspa4l)** | **95178** | **5.54** | **+** |
| **13** | **Gamma-actin (Actg1)** | **41335** | **5.56** | **+** |
| **14** | **Protein disulfide-isomerase-like protein of the testis precursor (Pdilt)** | **68002** | **5.57** | **+** |
| **15** | **Pyruvate dehydrogenase (lipoamide) beta (Pdhb)** | **35156** | **5.63** | **-** |
| **16** | **Heat shock protein 1-like: Hspa1l (Hsc70t)** | **70978** | **5.81** | **-** |
| **17** | **Ubiquinol-cytochrome c reductase core protein 1 (Uqcrc1)** | **53446** | **5.81** | **+** |
| **18** | **T-complex protein 1 (Tcp1)** | **60867** | **5.82** | **+** |
| **19** | **Unnamed protein product (Hsp70 related protein)** | **60878** | **6.04** | **+** |
| **20** | **PRP19/PSO4 pre-mRNA processing factor 19 homolog: S. cerevisiae (Prpf19)** | **55661** | **6.14** | **+** |
| **21** | **Fructose bisphosphatase 1 (Fbp1)** | **37288** | **6.15** | **-** |
| **22** | **Eukaryotic translation elongation factor 1 gamma (Eef1g)** | **50371** | **6.31** | **+** |
| **23** | **Phosphoglycerate kinase 2 (Pgk2)** | **45223** | **6.36** | **+** |
| **24** | **Leucine rich repeat containing 34 (Lrrc34)** | **47118** | **6.38** | **-** |
| **25** | **Unnamed protein product (EIF-4A related protein)** | **42386** | **6.56** | **+** |
| **26** | **Succinate-Coenzyme A ligase, ADP-forming, beta subunit (Sucla2)** | **50424** | **6.57** | **+** |
| **27** | **Aspartyl aminopeptidase isoform b (Dnpep)** | **52744** | **6.82** | **-** |
| **28** | **Me1 protein (Me1)** | **64426** | **6.87** | **+** |
| **29** | **Succine dehydrogenase Fp subunit (Sdha)** | **73623** | **7.06** | **+** |
| **30** | **Mitochondrial aldehyde dehydrogenase 2 (Aldh2)** | **57015** | **7.53** | **+** |
| **31** | **Dihydrolipoamide dehydrogenase (Dld)** | **54748** | **7.97** | **+** |
| **32** | **Glyceraldehyde-3-phosphate dehydrogenase, spermatogenic (Gapdhs)** | **48096** | **8.17** | **-** |
| **33** | **Heterogeneous nuclear ribonucleoprotein L (Hnrnpl)** | **64550** | **8.33** | **+** |
| **34** | **Pyruvate dehydrogenase E1 alpha 2 (Pdha2)** | **44183** | **8.79** | **-** |
| **35** | **DAZ-associated protein 1 (Dazap1)** | **43281** | **8.88** | **-** |
| **36** | **Unnamed protein product (Fumarate hydratase related protein)** | **54550** | **9.12** | **-** |
|  |  |  |  |  |

Supplementary Table S2

List of plasmid vectors (pCMV6-Entry)

| PDHA2 (Myc-DDK-tagged) - Mouse pyruvate dehydrogenase E1 alpha 2 (PDHA2) | MR215676 |
| --- | --- |
| FBP1 (Myc-DDK-tagged) - Mouse fructose bisphosphatase 1 (FBP1) | MR205054 |
| ATP6V1A (Myc-DDK-tagged) - Mouse ATPase, H+ transporting, lysosomal V1 subunit A (ATP6V1A) | MR209449 |
| LRRC34 (Myc-DDK-tagged) - Mouse leucine rich repeat containing 34 (LRRC34) | MR220486 |
| TUBB4B (Myc-DDK-tagged) - Mouse tubulin, beta 2C (TUBB2C) | MR207096 |
| HSPA1L (Myc-DDK-tagged) - Mouse heat shock protein 1-like (HSPA1L) | MR223206 |
| PDHB (Myc-DDK-tagged) - Mouse pyruvate dehydrogenase (lipoamide) beta (PDHB), nuclear gene encoding mitochondrial protein | MR205484 |
| DNPEP (Myc-DDK-tagged) - Mouse aspartyl aminopeptidase (DNPEP), transcript variant 2 | MR207553 |
| DAZAP1 (Myc-DDK-tagged) - Mouse DAZ associated protein 1 (DAZAP1), transcript variant 1 | MR215857 |
| GAPDHS (myc-DDK-tagged) – Mouse glyceraldehyde-3-phosphate dehydrogenase, spermatogenic (GAPDHS), transcript variant 1 | MR229930 |
| pCMV6-Entry, mammalian vector with C-terminal Myc- DDK Tag, 10ug | PS100001 |

Supplementary Figure S1

Immunohistochemical stain of 3β-HSD (a, c) and GATA1 (b, d) in the normal TGC (Testicular Germ Cell: c, d) and testis (a, b). 3β-HSD and GATA1 were detected in Leydig and Sertoli cells of the testis, but not in TGC. Broun dots were positive cells. Bar=100μm.


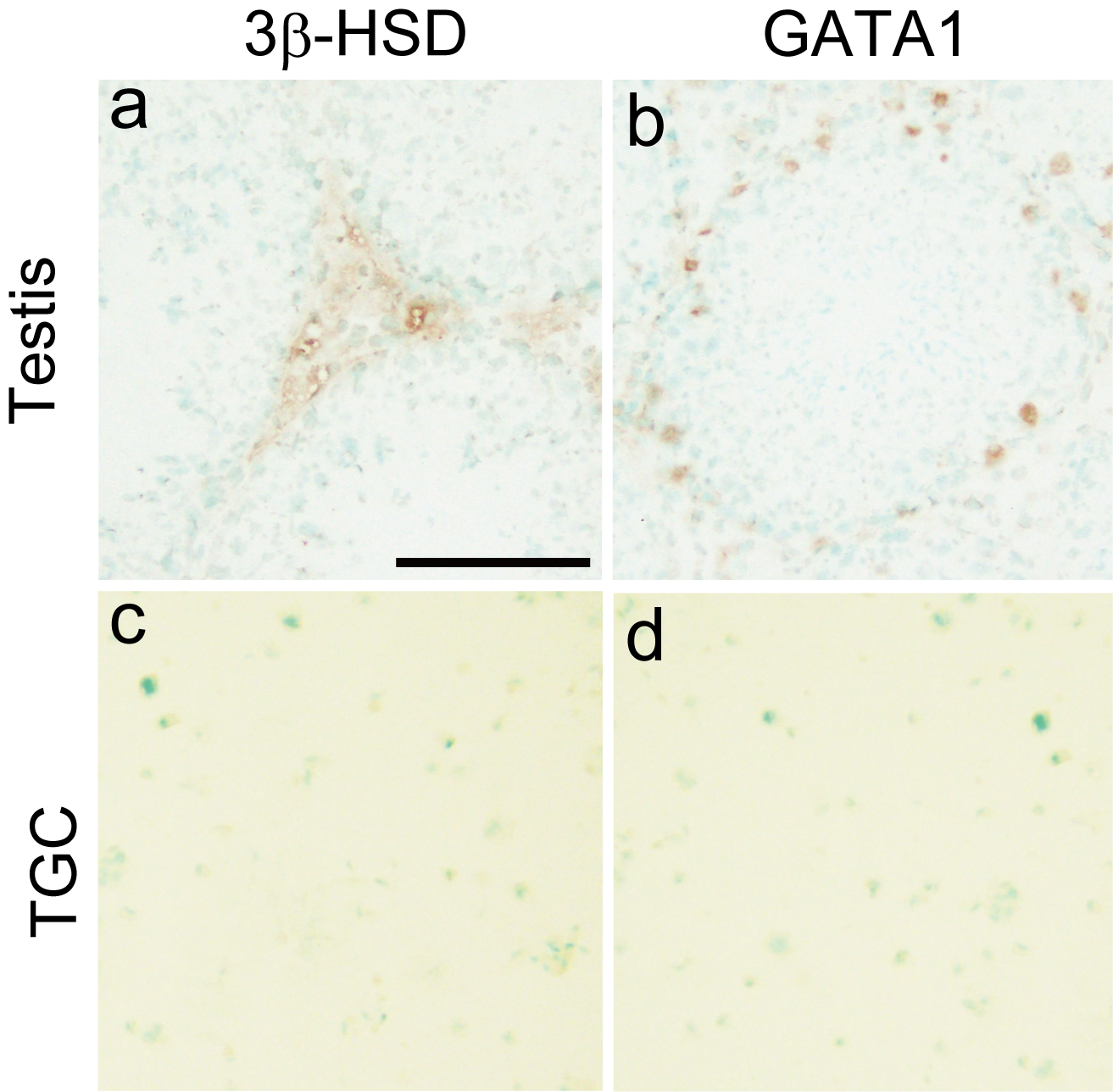


The testes and TGC of experimental mice were put in OCT compound (Miles Laboratories, Naperville, IL, USA), and then frozen in liquid nitrogen and stored at 80℃ until used. Sections of 5μm were cut with a cryostat (CM1900; Leica, Wetzlar, Germany), dried in air, and then fixed in 95% ethanol for 10 min at -20℃. The sections were rinsed in PBS and then incubated with Block Ace (Yukijirushi, Hokkaido, Japan) for 20 min at room temperature to inactivate endogenous peroxidase activity. After this treatment, serial sections were incubated for 2 hours with rat anti-mouse 3b-HSD antibodies (Leydig cell marker; Santa Cruz, USA, 1:200 dilution), rat anti-mouse GATA1 antibodies (Sertoli cell marker; Santa Cruz, USA, 1:200 dilution). After washing, the sections were incubated for 30 min with rabbit-anti rat IgG (Vector, CA, USA). Immuno-reactive cells were visualized with a Vectastain ABC Kit (Vector, CA, USA) with 0.05% 3, 3- diaminbenzidine 4HCl (DAB, Nickel Solution) and 0.01% H_2_O_2_ as the chromogen. The sections were finally counterstained with methyl green. Sections processed with PBS instead of the primary antibodies were used as negative controls.
